# Addressing Biodisaster X Threats With Artificial Intelligence and 6G Technologies: Literature Review and Critical Insights

**DOI:** 10.2196/26109

**Published:** 2021-05-25

**Authors:** Zhaohui Su, Dean McDonnell, Barry L Bentley, Jiguang He, Feng Shi, Ali Cheshmehzangi, Junaid Ahmad, Peng Jia

**Affiliations:** 1 Center on Smart and Connected Health Technologies, Mays Cancer Center School of Nursing UT Health San Antonio San Antonio, TX United States; 2 Department of Humanities Institute of Technology Carlow Carlow Ireland; 3 Cardiff School of Technologies, Cardiff Metropolitan University Cardiff United Kingdom; 4 Centre for Wireless Communications University of Oulu Oulu Finland; 5 Department of Research and Development Shanghai United Imaging Intelligence Shanghai China; 6 Faculty of Science and Engineering University of Nottingham Ningbo China Ningbo China; 7 Network for Education and Research on Peace and Sustainability Hiroshima University Hiroshima Japan; 8 Prime Institute of Public Health Peshawar Medical College Peshawar Pakistan; 9 Department of Land Surveying and Geo-Informatics The Hong Kong Polytechnic University Hong Kong China; 10 International Institute of Spatial Lifecourse Epidemiology Hong Kong China

**Keywords:** 6G, artificial intelligence, biodisaster X, biodisasters, biosafety, biosurveillance, biotechnology, bioterrorism, COVID-19, disease X, sixth-generation technologies

## Abstract

**Background:**

With advances in science and technology, biotechnology is becoming more accessible to people of all demographics. These advances inevitably hold the promise to improve personal and population well-being and welfare substantially. It is paradoxical that while greater access to biotechnology on a population level has many advantages, it may also increase the likelihood and frequency of biodisasters due to accidental or malicious use. Similar to “Disease X” (describing unknown naturally emerging pathogenic diseases with a pandemic potential), we term this unknown risk from biotechnologies “Biodisaster X.” To date, no studies have examined the potential role of information technologies in preventing and mitigating Biodisaster X.

**Objective:**

This study aimed to explore (1) what Biodisaster X might entail and (2) solutions that use artificial intelligence (AI) and emerging 6G technologies to help monitor and manage Biodisaster X threats.

**Methods:**

A review of the literature on applying AI and 6G technologies for monitoring and managing biodisasters was conducted on PubMed, using articles published from database inception through to November 16, 2020.

**Results:**

Our findings show that Biodisaster X has the potential to upend lives and livelihoods and destroy economies, essentially posing a looming risk for civilizations worldwide. To shed light on Biodisaster X threats, we detailed effective AI and 6G-enabled strategies, ranging from natural language processing to deep learning–based image analysis to address issues ranging from early Biodisaster X detection (eg, identification of suspicious behaviors), remote design and development of pharmaceuticals (eg, treatment development), and public health interventions (eg, reactive shelter-at-home mandate enforcement), as well as disaster recovery (eg, sentiment analysis of social media posts to shed light on the public’s feelings and readiness for recovery building).

**Conclusions:**

Biodisaster X is a looming but avoidable catastrophe. Considering the potential human and economic consequences Biodisaster X could cause, actions that can effectively monitor and manage Biodisaster X threats must be taken promptly and proactively. Rather than solely depending on overstretched professional attention of health experts and government officials, it is perhaps more cost-effective and practical to deploy technology-based solutions to prevent and control Biodisaster X threats. This study discusses what Biodisaster X could entail and emphasizes the importance of monitoring and managing Biodisaster X threats by AI techniques and 6G technologies. Future studies could explore how the convergence of AI and 6G systems may further advance the preparedness for high-impact, less likely events beyond Biodisaster X.

## Introduction

Humans have been living with disasters for thousands of years. Records from ancient civilizations, albeit difficult to come across and piecemeal, document the undeniable presence and the lingering impacts of disasters throughout human history [1-8]. Evidence from recent decades alone, for instance, shows that the world witnesses approximately 400 natural disasters and 30-40 armed conflicts annually [9]. Although already daunting, these numbers likely underrepresent the accurate scale and severity of these disasters in society; many often occur in remote places that draw little to no attention of the media or the research community. While humanity is no stranger to various disaster-led debates and discussions focused on the risks posed by natural and anthropogenic disasters [10-12], collectively, research still indicates that societies at large perform poorly when it comes to disaster preparedness [13-19].

As apparent from the COVID-19 pandemic, from the measures assembled during the first outbreaks in 2020 to the faltering waves of public health policies established throughout the first months of 2021, the lack of disaster preparedness and readiness in the wake of global health crises resulted in drastic consequences to the economy and to society [20-25]. Even though the World Health Organization made Disease X (a placeholder term for any unknown pathogenic disease with a pandemic potential) a global priority in 2018 [26], and warned of the potential for a COVID-19–like pandemic to cause disorder in society, unfortunately, few measures were taken to prepare for it [27]. As of May 6, 2021, approximately 155 million COVID-19 cases and over 3.24 million mortalities have been reported worldwide [28]. Parallel with the growth of scientific research in the biological sciences, warnings of the importance of pandemic preparation have been issued throughout the twentieth century [26,29-33] across multiple disciplines. The possibility of bioterrorism and a pathogen’s potential to be unleashed without countermeasures is an increasing concern [34-36]. The use of bioweapons by state actors has long been a risk [37-45]; however, with rapid developments in biotechnology, it becomes increasingly feasible for nonstate actors to develop powerful bioweapons in low-barrier contexts, such as home environments, thus increasing the likelihood of biodisasters [46].

Overall, following the pattern of “Disease X,” here we introduce the term “Biodisaster X” to refer to disasters caused by the accidental or intentional misuse of biotechnologies by state or nonstate actors. In general, there are no practical insights on approaches to monitor and manage Biodisaster X preemptively. Considering the potential risks Biodisaster X could pose to society’s safety and security, research is urgently needed to bridge the knowledge gap. Advances in information and communication technologies have revealed several opportunities to harness digital tools in this effort, particularly with the latest generation of artificial intelligence (AI) techniques and the development of 6G wireless communication technologies, which arguably includes the most promising advanced technological platforms for addressing Biodisaster X threats. Therefore, to this end, this study aimed to identify (1) the potential dangers Biodisaster X poses to society at large and (2) solutions that use emerging 6G and AI technologies to help monitor and manage Biodisaster X threats.

## Methods

### Methods Overview

A review of the literature on the application of AI and 6G technologies for monitoring and managing biodisasters was conducted via PubMed, using articles published from database inception through to November 16, 2020. The literature search focused on three themes: AI techniques, 6G technologies, and biodisasters. The search terms we utilized are listed in Table 1. To ensure that up-to-date evidence was included, validated news reports, articles identified by examining the reference lists of eligible articles, and records found through an updated literature search in January 2021 were also included.

**Table 1:** Search terms used on PubMed.

Theme	Search term
Artificial intelligence	“artificial intelligence”[MeSH^a^] OR “artificial intelligence”[TIAB^b^] OR “machine learning”[MeSH] OR “machine learning”[TIAB] OR “deep learning”[MeSH] OR “deep learning”[TIAB]
6G	“sixth-generation communication*”[MeSH] OR “sixth-generation communication*”[TIAB] OR “sixth-generation network*”[MeSH] OR “sixth-generation network*”[TIAB] OR “sixth-generation technolog*”[MeSH] OR “sixth-generation technolog*”[TIAB] OR “sixth-generation cellular” [MeSH] OR “sixth-generation cellular” [TIAB] OR “6G communication*”[MeSH] OR “6G communication*”[TIAB] OR “6G network*”[MeSH] OR “6G network*”[TIAB] OR “6G technolog*”[MeSH] OR “6G technolog*”[TIAB] OR “6G wireless”[MeSH] OR “6G wireless”[TIAB] OR “6G cellular”[MeSH] OR “6G cellular”[TIAB]
Biodisasters	biodisaster*[MeSH] OR biodisaster*[TIAB] OR bio-disaster*[MeSH] OR bio-disaster*[TIAB] OR bioterror*[MeSH] OR bioterror*[TIAB] OR “biological warfare”[MESH] OR “biological warfare”[TIAB] OR “chemical warfare”[MeSH] OR “chemical warfare”[TIAB] OR “bacterial infections and mycoses”[MeSH] OR “bacterial infections and mycoses”[TIAB] OR “virus disease*”[MeSH] OR “virus disease*”[TIAB] OR “parasitic disease*”[MeSH] OR “parasitic disease*”[TIAB] OR “biological threat*”[MeSH] OR “biological threat*”[TIAB] OR bioterror* OR biowar*

^a^MeSH: Medical Subject Headings.

^b^TIAB: limit to title or abstract.

### Inclusion and Exclusion Criteria

The literature inclusion criteria for studies in this literature review are listed in Table 2. Overall, articles were excluded if they (1) were not published in English, (2) did not focus on biodisasters, (3) did not focus on either AI techniques or 6G technologies, or (4) did not provide detailed information on the utilization of AI techniques or 6G technologies for monitoring or managing biodisaster threats.

**Table 2:** Study inclusion criteria.

Data type	Inclusion criteria
Language	English
Study context	Centered on discussions about biodisasters (disasters that occur as a result of infectious pathogens with bioweapon potentials unleashed by state or nonstate actors)
Technology type	Artificial intelligence and 6G
Study design	Focus on utilizing artificial intelligence techniques or 6G technologies to address issues associated with biodisasters

## Results

Drawing insights from the review of the literature, the broader literature, as well as the structure of our research objectives and overall study foundation, a clear and comprehensive comparison of the similarities and differences between Disease X and Biodisaster X was developed, details of which are provided in Table 3. As our research objectives were focused on the real-world impact and implications of Disease X and Biodisaster X, rooted in, yet above and beyond what has been discussed and debated in the current literature, our results have been arranged as such. Additionally, to provide a structured and systematic understanding of the findings, we further modeled the study results using the following frames: type of disaster, origin, antecedent, pathogen, transmissibility, transmission predictability, controllability and treatability, nonpharmaceutical mitigation effort, pharmaceutical solution, primary goal, as well as positive unanticipated outcome and negative unintended consequence. A detailed comparison of Disease X and Biodisaster X has been made in Table 3.

**Table 3:** Similarities and differences between Disease X and Biodisaster X.

Parameter	Disease X	Biodisaster X
Type of Disaster	Infectious diseases Natural disasters: epidemics or pandemics	Infectious diseases Anthropogenic biodisasters: initially inaccurately identified as a naturally occurring epidemic or pandemic; later identified as anthropogenic
Origin	None initially, but can be exacerbated by human action or inaction; for example, through livestock mismanagement or failure to contain early infections	Humans: state or nonstate actors
Antecedent	None	Advances in and accessibility of biotechnology; inadequacy of biosecurity measures; intent or malice of nonstate actors
Pathogen	Originated in nature, with no human engineering Unfamiliar or unknown to humans	Originated in a laboratory, mainly as a result of human engineering Unfamiliar or unknown to humans
Transmissibility	Highly transmissible, mainly as a result of naturally occurring human interconnectivity	Highly transmissible, mainly as a result of calculated dissemination and distribution of the pathogen, capitalized on naturally occurring human interconnectivity
Transmission Predictability	Initially unpredictable; predictability increases over time	Initially predictable (in principle); extremely low to extremely high predictability over time (depending on the ability of society at large to identify the risk)
Controllability and Treatability	Low	Extremely low to extremely high (based on the intent of the actor)
Nonpharmaceutical Mitigation Effort	Have an agile, evidence-based, and flexible disaster response plan Equip high-population-density areas with sufficient resources Limit social interactions; replace physical human interconnectivity with web-based social interactions when possible Integrate cost-effective technology-based surveillance systems into the emergency management systems Prepare for a secondary Disease X (potential for mutations or gene transfer, in turn leading to a new Disease X)	Have an agile, evidence-based, and flexible disaster response plan Equip high-population density areas with sufficient resources Limit social interactions; replace physical human interconnectivity with web-based social interactions when possible Integrate cost-effective technology-based surveillance systems into the emergency management systems Prepare for a secondary Biodisaster X (actors might further escalate the situation by generating a new Biodisaster X). Once accurately identified as anthropogenic in nature, there will be an urgent need prioritize the identification of the actor
Pharmaceutical Solution	Difficult and time-consuming to develop vaccines Extensive and exhaustive efforts needed to identify treatment plans	Have the potential to be developed fairly easily and time-efficiently once the nonstate actors and the pathogen manufacture process have been identified: vaccine and treatment plans
Primary Goal	Stop the spread of the pathogen; identify and deploy suitable treatments	Stop the spread of the pathogen; identify and deploy suitable treatments; locate the source of the pathogen and prevent further action
Positive Unanticipated Outcome and Negative Unintended Consequence	Physical and psychological health issues associated with the pharmaceutical and nonpharmaceutical solutions Posttraumatic stress Increased resource allocation for medicine and public health Possible economic consequences including a global recession	Physical and psychological health issues associated with the pharmaceutical and nonpharmaceutical solutions Posttraumatic stress Increased resource allocation for medicine, public health, and law enforcement Possible economic consequences including a global recession

## Discussion

Biodisaster X could result in catastrophic human and economic consequences. Yet to date, no studies have examined the potential role of information technologies in preventing and mitigating Biodisaster X, especially in the context of advanced and emerging technologies such as AI and 6G. To bridge the research gap, in this study, we sought to explore 2 fundamental research questions that could considerably enrich the literature: (1) what Biodisaster X might entail and (2) solutions that use AI and emerging 6G technologies to help monitor and manage Biodisaster X threats. In the following sections, we will detail our findings pivoting on these 2 research objectives, as well as practical and powerful strategies that have the potential to effectively control and contain Biodisaster X threats.

### Biodisaster X: What is in a Name?

A disaster can be defined as “a serious disruption of the functioning of a community or society involving widespread human, material, economic, or environmental losses and impacts, which exceeds the ability of the affected community or society to cope using its own resources” [47]. Based on the contributing causes, disasters are usually categorized as natural (eg, earthquakes, infectious disease-inducing epidemics, or pandemics of natural origin) and anthropogenic (eg, armed conflicts, nuclear accidents, or the release of pathogenic genetically modified organisms from laboratory settings). In the context of this study, biodisasters are defined as disasters that occur as a result of infectious pathogens with bioweapon potential, which are unleashed by state or nonstate actors accidentally and intentionally (eg, the Japanese government’s controversial decision to dump Fukushima’s contaminated water into the boundless and borderless ocean shared by all life forms on earth, including humans and sharks [48]). In the context of biodisasters, a state actor often takes the form of a nation that deliberately and systematically designs and develops infectious pathogens with its national interest in mind. In contrast, a nonstate actor is an individual or group acting independently to obtain or manufacture a pathogen either owing to misguidance or malice. Of note, although existing multilateral agreements prohibit the production and use of bioweapons by state actors (termed biowarfare) [49], the presence of signed agreements does not imply that accidental or intentional development and release of pathogens by state actors will not occur.

The concept of “bioterrorism,” defined as the deliberate release of pathogens that could cause illnesses and deaths in society, is not the focus of this study because “bioterrorism” entails both deliberation and malice (eg, to elicit terror to the public) [50]; antecedents may not necessarily apply to Biodisaster X threats. Insights from behavioral science [51-53] and evidence regarding individual-caused mass casualty events (eg, indiscriminate mass shootings) [54-56] suggest that individual actors’ behaviors, potentially leading to the onset of Biodisaster X, may or may not include conscious deliberation to harm. In other words, while it is possible that individual actors’ malicious actions might cause some biodisasters, it is also possible that some individual-caused biodisasters are accidental.

Furthermore, the term bioterrorism is limited, in that “terror” is the main outcome. We believe that for Biodisaster X, which could upend lives, livelihoods, and economies, “disaster” is a more appropriate description that sheds light on the scale and severity of its consequences and is more diverse than “terror.” Drawing insight from real-world examples, similar to the prevalent ransomware hacks, it is possible that state or individual actors could develop and utilize infectious pathogens as “ransomgens” for financial gain rather than merely aiming to generate terror in society. Therefore, under the current research context, we adopted the term “biodisaster” instead of “bioterrorism.” Furthermore, considering that various studies have discussed approaches to address state actor–initiated biodisasters [57-61], this study focuses on biodisasters that are infectious in nature, caused by individual actors, and can result in catastrophic human and economic consequences.

### Biodisaster X vs Disease X

The risk of biodisasters, such as Biodisaster X, is increasing in likelihood: advances in technology, particularly the availability and maturity of biotechnology, have grown considerably in recent years. Inadvertently, these advances may resemble those of Oppenheimer [62] in facilitating the release of destructive factors. One example of the misuse of biotechnology is a microbiologist, vaccinologist, and senior biodefense researcher who worked at the United States Army Medical Research Institute of Infectious Diseases, who allegedly engineered the 2001 anthrax attacks [63-65]. While the scale of the 2001 anthrax attacks was minor, it demonstrated how easily biodisasters can occur and how unprepared society was for these events. As seen in the lack of adequate preparation and coherent responses to infectious disease–induced pandemics, including COVID-19 [66-69], Biodisaster X’s effects may be compounded to the same, if not greater, degree by incompetence across international, national, and regional agencies and organizations.

The concept of Biodisaster X can be best understood in contrast with Disease X. In terms of similarities, both Biodisaster X and Disease X are driven by pathogens unknown to humans and have the potential to cause crippling effects on society. Furthermore, based on previous inadequacies in response to emergency events including pandemics [66-74], the world at large may be ill-prepared for both Biodisaster X and Disease X. In terms of unique attributes, compared to Disease X, Biodisaster X is more likely to have the following characteristics: (1) having a pathogen directly affiliated to a laboratory; (2) having distinctive and engineered attributes tailored by the capabilities and intentions of the developer; and (3) the origin, development, and history can be definitively ascertained upon identification of the developer, which is not possible for naturally occurring pathogens (eg, the 1918 influenza pandemic), where there is always uncertainty regarding the origin and evolutionary history of the disaster [75-77].

### The Imperative of Preparing for Biodisaster X

Some of the deadliest pandemics—the most recent ones ranging from AIDS, severe acute respiratory syndrome, Middle East respiratory syndrome, Ebola, and COVID-19—all have zoonotic origins [78]. Studies have further shown that for viruses that can transmit from animals to humans, especially those that can infect a diverse range of host species, the transmission speeds are substantially amplified once human-to-human transmission is established, and the diseases can quickly evolve into global pandemics [79]. Consequently, once a pathogen is transmissible within a population, there is a low access threshold: an individual actor can “obtain” these deadly pathogens without the need for advanced laboratory skills or extensive financial resources. However, costs to physical and mental health may reveal a counternarrative.

Based on available evidence, it is difficult to determine whether an individual can be a malicious “patient zero”; an individual who intentionally contracts a novel virus intending to cause infectious disease outbreaks in a society [80]. It is not impossible to purposely study and capture known or unknown deadly pathogens that can trigger infectious diseases; microbial surveys are commonly conducted to identify novel pathogens before they pose a threat to public health [81-84]. In theory, there could be individual actors, with adequate knowledge or experience (similar to the microbiologist allegedly behind the 2011 anthrax attacks [63-65]), who may take the same actions but with different motives, ranging from scientific curiosity to ill-guided intentions. Considering the rich biodiversity of wildlife, along with the large number of “missing viruses” and “missing zoonoses” that remain unidentified [85], close contacts with latent deadly pathogens are nearly impossible to control, which in turn, renders it challenging to locate or identify individual actors who might utilize them. Advances in synthetic biology may further compound the situation, especially considering the scholarly endeavors using pathogens in laboratory settings, which could amount to the level of real-world pandemics (eg, laboratory-cultured viruses such as smallpox [86-88]). The likelihood of Biodisaster X increases in proportion to these factors.

Overall, considering the species diversity of wildlife, the unknown factors related to the scale and severity of viruses in animals, which have the latent potential to infect humans, and the varying degrees of competency of community health centers in detecting infectious disease outbreaks in a bottom-up manner, it could be tremendously difficult for health experts and government officials to monitor potentially emerging Biodisaster X threats. However, not all hope is lost. Technology-based solutions, especially those utilizing AI and 6G technologies, can help address these issues.

### The Need for Advanced Technology Solutions for Monitoring and Managing Biodisaster X

#### The Need for Technology-Based Solutions

Once Biodisaster X becomes a reality, human contact will drive transmission and become the primary fuel for exacerbating infections and deaths caused by the disaster. As seen during the COVID-19 pandemic, owing to virus spread and subsequent public health policies (eg, lockdowns), many critical societal functions could be substantially disrupted. The potential to control and contain human and economic consequences of Biodisaster X, such as the functionality of the health care systems (eg, infected health care professionals) [89-91], may also become critically undermined. In these circumstances, technology-based solutions could be the key to addressing these crises, as they are different from conventional solutions; they are not highly dependent on physical interactions and transportation. Overall, technology-based solutions require limited human resources (eg, with the ability to operate without human input), can be delivered independent of physical human contact (eg, web-based and remote deployment), and are immune to infectious diseases (eg, can function in contaminated environments). Furthermore, technology-based solutions are less vulnerable to issues ranging from physical fatigue to mental health burdens, which are health challenges that frontline workers often face amid emergency events.

#### The Need for Advanced Technologies

To effectively predict, control, and manage Biodisaster X, which is an event with a low probability (ie, difficult to detect preemptively) and a high impact (ie, difficult to control and contain), advanced technologies are needed. While many emerging technologies can address the dangers and damages associated with Biodisaster X [92,93], 2 families of advanced technology-based solutions show particular promise, namely AI techniques and 6G technologies.

#### Unique Capabilities of AI

AI is generally considered synonymous with “thinking machines” [94], or techniques that can facilitate “a computer to do things which, when done by people, are said to involve intelligence” [95]. With AI technologies, machines can identify patterns too intricate for humans to identify and process quickly. AI techniques are widely used in areas such as natural language processing, speech recognition, machine vision, targeted marketing, and health care, including efforts to combat COVID-19 [96-99]. While technologies such as virtual reality, smart sensors, drones, and robotics could play a positive role in supporting health care professionals to cope with the pandemic [100-102], AI technologies are arguably most instrumental in addressing some of the most prominent issues health experts and government officials are faced with, ranging from pandemic surveillance to COVID-19 drug and vaccine development [103-106].

AI and machine learning techniques are particularly valuable in their ability to identify trends and patterns across large amounts of data promptly and cost-effectively; for example, in identifying or searching for specific patterns. With natural language processing, for instance, data can be extracted retrospectively from clinical records or prospectively in real time and statistically processed for insights, which, in turn, can supplement existing structured data to enrich actionable information [86]. During the COVID-19 pandemic, natural language processing models have been used to analyze publicly available information such as tweets, tweet timestamps, and geolocation data, to identify and map potential COVID-19 cases cost-effectively, without utilizing testing devices or other medical resources that involve health care professional [107].

Overall, most, if not all, AI techniques are irreplaceable in regard to administering complex tasks such as extracting useful information from large data sets. Moreover, with the continuously increasing speed of its technological advancements and applications, AI technologies are often utilized as core components in other emerging technologies [108]. Smart sensors that perform advanced tasks, such as effectively identifying and recognizing captured motions and images, often need to integrate deep learning technologies (a subgroup of AI) [109-111]. These combined insights suggest that AI techniques have great potential in monitoring and managing Biodisaster X threats.

#### Unique Capabilities of 6G Networks

6G technologies are the next generation of wireless communication systems following 5G networks [112]. While 6G is still under development, it is envisioned as the most capable communication network currently available [112-119]. The advantages of 6G networks derive from their high data transmission speed (up to 1 terabyte per second), wireless hyper-connectivity (100 million connections per km^2^), low end-to-end latency (< 1 ms), reliability (1-10^-9^) (reliability in terms of the frame error rate, which is defined as the ratio of the number of incorrectly decoded frames to that of total transmitted frames), and high-accuracy positioning capabilities (indoor: <10 cm in 3D; outdoor: <1 m in 3D) [112-119]. Adding the fact that 6G networks also excel in their energy efficiency and spectrum efficiency, these networks can provide fast and efficient wireless reporting and access to remote computational facilities, facilitating mobile biomonitoring and disaster management.

**Figure 1:**
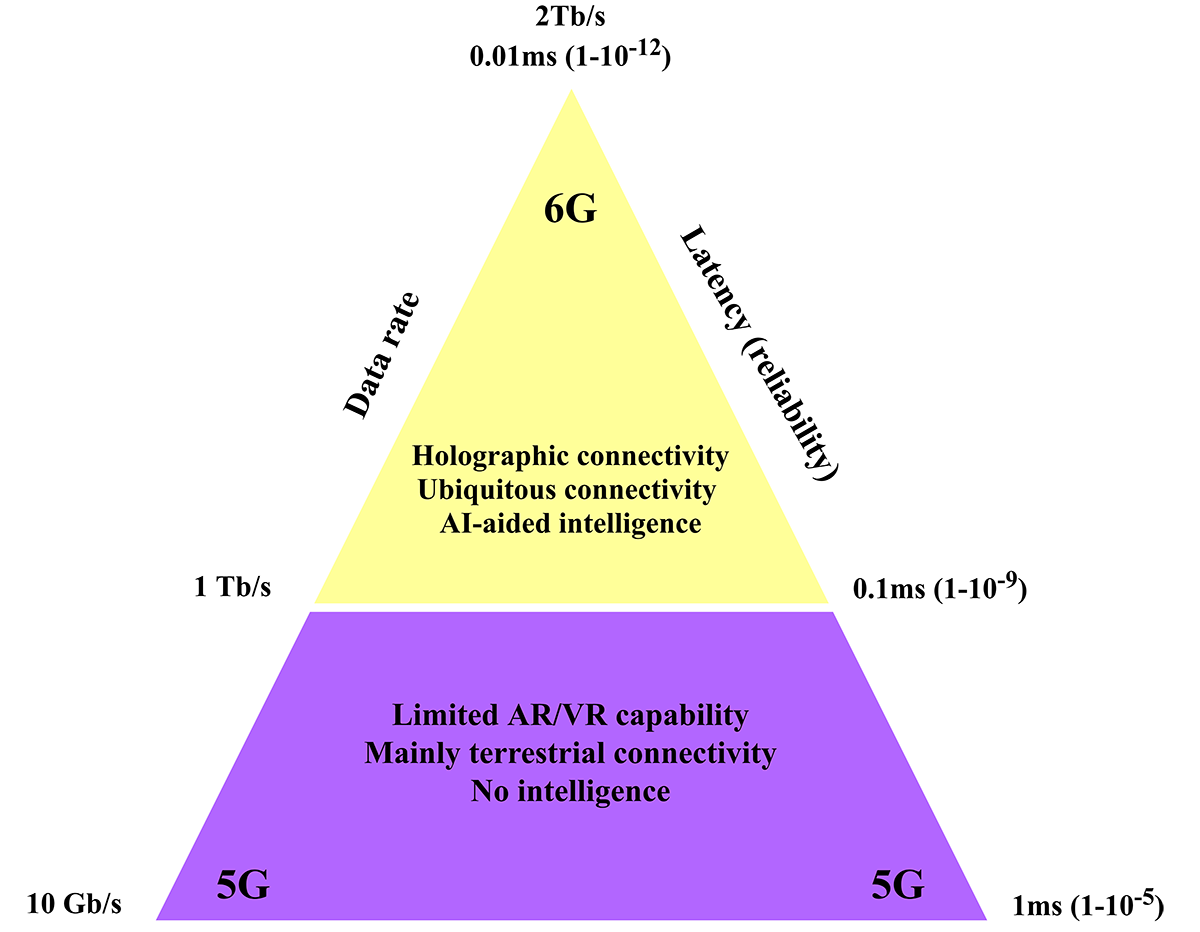
A schematic representation of the unique advantages of 6G compared to 5G technologies. AI: artificial intelligence, AR: augmented reality, VR: virtual reality.

For instance, the high reliability and data transmission speed of 6G technologies will be of critical importance amid global emergency events with the scale of Biodisaster X. At the onset of the COVID-19 pandemic, many internet companies and service providers experienced outrage and were forced to reduce the amount of data individuals and organizations could utilize to ensure continuous communication for all [120]. This limitation of existing communication networks could compromise the ability of health experts and government officials to monitor and manage COVID-19–related threats and other disasters promptly and properly. Of note, in the face of an extremely deadly, contagious, and fast-developing Biodisaster X, information will be predominantly updated and exchanged remotely and over the internet. The speed and success of updating and exchanging information are highly dependent on the reliability of communication networks, in which 6G technologies excel, especially when spatial big data have been introduced for disease control and prevention since the COVID-19 pandemic [27,108,121]. Figure 1 lists visual comparisons in communication capabilities between 6G and 5G networks.

### AI and 6G Technologies for Biodisaster X Control and Management

**Figure 2:**
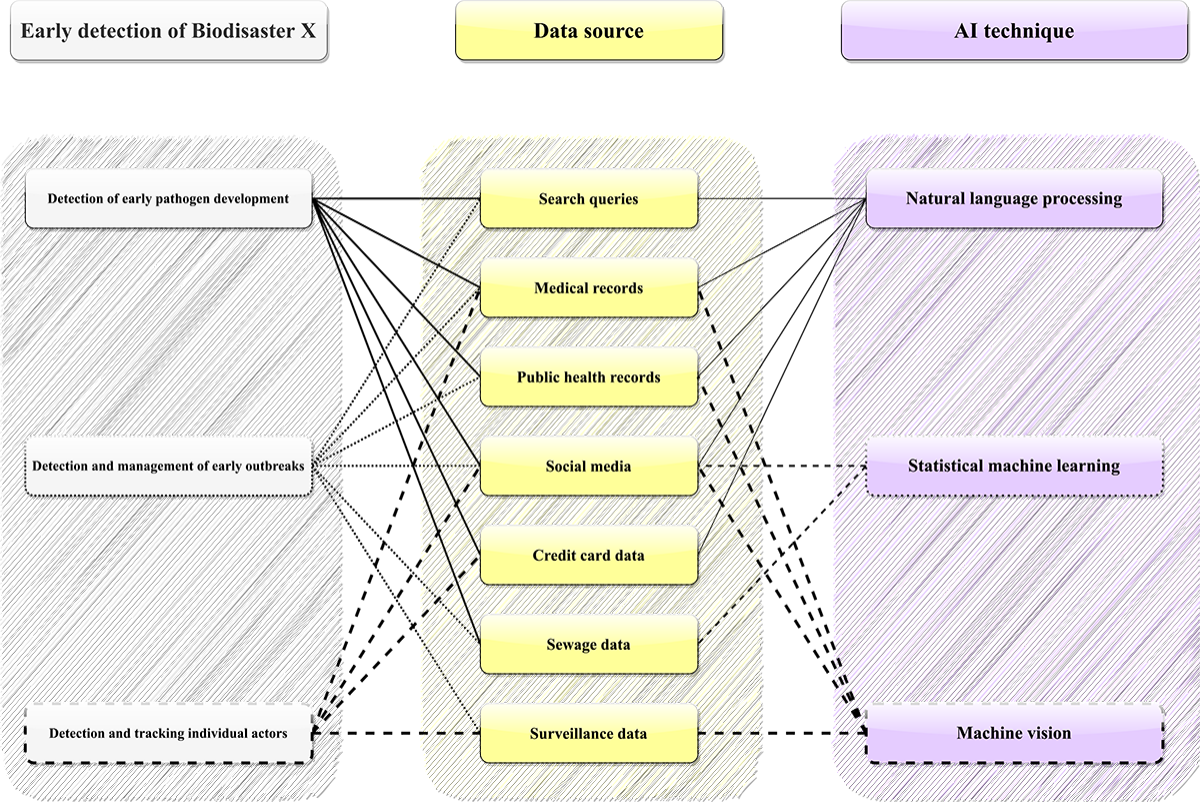
Artificial Intelligence techniques discussed in detail in the study. Statistical machine learning refers to techniques that do not involve natural language processing or deep learning processes. AI: artificial intelligence.

Drawing insights from the COVID-19 pandemic [103-106], AI techniques, especially when coupled with advanced communication capabilities enabled by 6G technologies, can elevate biodisaster control and management. In other words, 6G-based AI technologies can be applied to address issues ranging from early Biodisaster X detection (eg, identification of suspicious behaviors) to remote design and development of pharmaceuticals (eg, treatment development) and public health interventions (eg, reactive shelter-at-home mandate enforcement), and disaster recovery (eg, sentiment analysis of social media posts to shed light on the public sentiments and readiness for recovery building). While there are research issues worth exploring, in this study, we specifically (1) focus on early detection of Biodisaster X, a disaster management stage that could yield maximum benefit in personal and public health protection, and (2) discuss critical aspects of the utilization and application of 6G-based AI technologies in monitoring and managing Biodisaster X threats. Further information on these AI techniques is available in Figure 2.

#### Natural Language Processing Analysis

With the benefit of high reliability and high data transmission speed, rather than depending on 1 source of data, 6G-based AI surveillance systems could synthesize various data sources to identify suspicious behaviors for epidemiologists, public health experts, and government officials for further analysis; this may also enable the National Intelligent Syndromic Surveillance System (an AI-based disease surveillance system) [108,122]. As available literature suggests [123-126], for instance, with the help of 6G technologies, natural language processing could be applied to analyze search queries (eg, searches such as “How to build an influenza virus weapon”), credit card transaction data (eg, receipts for equipment that are indispensable to develop a deadly virus), medical records (eg, whether the individual actor has a recent or frequent infectious disease diagnosis), public health records (eg, infectious disease cases coinciding with an actor’s activities), and social media activities (eg, reports of infectious disease symptoms and suspicious behaviors) interactively and in real time. While the analysis of isolated individual data sources may yield more noise than useful information, when the analyses are synthesized with corresponding data from diverse and complementary data sources, actionable information could become more readily available. 

It is important to note that natural language processing can also help shed light on people’s mental health, such as identifying at-risk populations with severe mental health disorders including schizophrenia and suicide attempts [127-129]. In an analysis of 826,961 unique Reddit users from 2018 to 2020, with the help of natural language processing techniques, researchers have been able to determine how specific mental health disorders (eg, schizophrenia) manifest in textual language, and, in turn, cost-effectively and unobtrusively identified at-risk mental health in social media users [130]. In the same study, natural language processing also offered researchers the opportunity to compare pre– and mid–COVID-19 posts to identify people whose mental health disorders had become more pronounced during the pandemic [130].

#### Statistical Machine Learning Solutions

To increase the usefulness of information, 6G-based AI systems can also capitalize on previously tested infectious disease control and management techniques, such as sewage-based epidemiology. Sewage-based epidemiology, or waste-based epidemiology, has helped scientists detect early warnings of pathogenic virus outbreaks, ranging from hepatitis A and norovirus to COVID-19 [131-135]. Different from conventional sewage monitoring, which incorporates little to no innovative technologies, the utilization and application of cost-effective and data-driven technologies in the field have significantly upscaled the information that researchers can gather from wastewater, thus reducing monitoring and reporting times, and, in turn, elevating societies’ ability to better monitor and manage infectious diseases [132-135]. The United Kingdom, for instance, has been rapidly developing its sewage monitoring systems to track COVID-19 outbreaks to understand pandemic transmission patterns more swiftly and accurately [136]. By analyzing human waste, researchers in the United Kingdom and worldwide were able to detect RNA from SARS-CoV-2 and better identify the location, scale, and possible trajectory of COVID-19 transmission [136-138].

However, current sewage monitoring technologies often face challenges such as delays in generating insights and inaccurate results owing to limitations caused by poor data processing speed [115,116], which could be overcome by 6G-based AI systems. The use of machine learning techniques has improved the ability to monitor events, such as poliovirus and enterovirus outbreaks with greater precision, as machine learning models could substantially improve surveillance sensitivity and the quantity and quality of actionable information garnered from wastewater [139]. Overall, with the high reliability and high data transmission speed provided by 6G networks, AI systems could advance sewage epidemiology (eg, through widespread remote analysis and evaluation) and facilitate real-time analyses of potential anomalies such as high volume or density of pathogenic viruses in waste and early infectious disease cases, before these threats progress to outbreaks. Insights gained from natural language processing can be further synthesized with information gained through other control measures, such as systematic and comprehensive sewage monitoring (eg, whether there is a suspicious presence of known or suspected pathogens in certain neighborhoods).

#### Deep Learning–Based Image Analysis

In addition to using natural language processing and statistical machine learning techniques, deep learning–based image analysis can also help with the early detection of Biodisaster X, particularly with the assistance of advanced networks enabled by 6G technologies. One application of deep learning in Biodisaster X monitoring and management would be gauging whether there are patterned disease outbreak signs, on the basis of insights gained from analyzing nontextual health records, such as medical images and real-time surveillance footage. While unstable and low-speed communication networks could affect AI-based disease surveillance architectures that use both regular reporting and remote computation systems, the influences of low-speed networks will be more pronounced for remote systems that use server-side analysis of high-resolution images to generate actionable information. This high network demand may create issues, such as requiring down-sampling of data, in turn compromising discrimination power and accuracy and the ability of the system to yield useful insights [140].

Machine vision enables machines to recognize visuals with the support of cameras, images, and learning models [141]. These processes can be fine-tuned to recognize and identify objects, such as patients’ medical images, and help health care professionals better gather information from imaging data and yield diagnoses and treatment plans [142-144]. Combined with the high speed of 6G networks, remote machine vision could be made more effective by allowing the transmission of high-quality images (eg, high resolution) and video-based surveillance (eg, no lag, high frame rate) in real time, facilitating more remote processing than would be possible otherwise.

With 6G technologies, deep learning–based machine vision could have “superhuman” capabilities in monitoring and managing Biodisaster X threats (eg, tracking multiple objects simultaneously and providing real-time analysis of potential threats with high accuracy), helping health officials track and monitor individual actors once identified. With advancements in 6G and deep learning techniques, machine vision may eventually have the potential to identify individual actors, even when they wear face masks. Scientists have proposed the use of machine vision to help health experts and government officials monitor and reinforce COVID-19 safety rules, such as face-making and social distancing (eg, masked face recognition) [145-147].

### Limitations

While this study addresses important knowledge gaps in the literature, there are some limitations to consider. First, owing to the scarcity of literature on the subject, we were unable to adopt a systematic review approach to investigate the research question. While our scoping search is justified for the scale of the current studies in this area, it nonetheless limits our findings. Second, while preparation plans for biodiaster X should involve multiple elements, ranging from surveillance, biosecurity mandates, and communication guidelines, to personnel training, we only discussed 1 aspect of Biodisaster X control and prevention. To address these limitations, assuming an increasing number of studies in this field, future studies should adopt a systematic or more comprehensive approach to examine this topic for further understanding ways to better prepare for Biodisaster X threats. Of note, although many AI techniques are now well-established, 6G technologies are still in their infancy. With the many unknown factors associated with AI and 6G development, it is possible that in the future, 6G and AI technologies may underperform in their ability to assist biodisaster monitoring and management, compared to our expectations and discussion in this study. However, this limitation may only be adequately addressed once empirical evidence on real-world 6G-based AI applications becomes more available and accessible.

### Conclusions

Biodisaster X is a looming but avoidable catastrophe. Considering the potential human and economic consequences Biodisaster X could have, actions that can effectively monitor and manage Biodisaster X threats must be taken promptly and adequately. Rather than solely depending on overstretched professional attention of health experts and government officials, it is perhaps more cost-effective and practical to deploy technology-based solutions to prevent and control Biodisaster X threats. This study discusses what Biodisaster X could entail and emphasizes the importance of monitoring and management of Biodisaster X threats through AI techniques and 6G technologies. Future studies could investigate how the convergence of AI and 6G systems may further advance the preparedness for high-impact, less likely events beyond Biodisaster X, including the facilitation of the development of the national intelligent syndromic surveillance system.

## Abbreviations

AI: artificial intelligence

## References

[1] Tang Q (2020). Ritual Healing: Records of Disasters and ritual function in Li Ji. Ritual Civilization and Mythological Coding.

[2] Touliatis PG (1996). Seismic Disaster Prevention in the History of Structures in Greece. Protection of the Architectural Heritage Against Earthquakes.

[3] Ciottone GR (2016). Introduction to Disaster Medicine. Ciottone's Disaster Medicine (2nd Edition).

[4] Noji EK (1997). The nature of disaster: general characteristics and public health effects. The Public Health Consequences of Disasters.

[5] Wagner DM, Klunk J, Harbeck M, Devault A, Waglechner N, Sahl JW, Enk J, Birdsell DN, Kuch M, Lumibao C, Poinar D, Pearson T, Fourment M, Golding B, Riehm JM, Earn DJD, Dewitte S, Rouillard J, Grupe G, Wiechmann I, Bliska JB, Keim PS, Scholz HC, Holmes EC, Poinar H (2014). Yersinia pestis and the plague of Justinian 541-543 AD: a genomic analysis. Lancet Infect Dis.

[6] Seger T (1982). The Plague of Justinian and Other Scourges: An analysis of the Anomalies in the Development of the Iron Age population in Finland. Fornvännen.

[7] Dols MW (1974). Plague in Early Islamic History. J Am Orient Soc.

[8] Wu T, Perrings C, Kinzig A, Collins JP, Minteer BA, Daszak P (2017). Economic growth, urbanization, globalization, and the risks of emerging infectious diseases in China: A review. Ambio.

[9] Waldman R (2018). Natural and human-made disasters. Centers for Disease Control and Prevention.

[10] Smith KF, Sax DF, Lafferty KD (2006). Evidence for the role of infectious disease in species extinction and endangerment. Conserv Biol.

[11] Matheny JG (2007). Reducing the risk of human extinction. Risk Anal.

[12] Leslie J (1996). The end of the world: The science and ethics of human extinction.

[13] Alexander DC (1993). Natural Disasters.

[14] Ritchie H, Roser M (2019). Natural disasters. One World in Data.

[15] Su Z, McDonnell D, Ahmad J (2021). The need for a disaster readiness mindset: A key lesson from the coronavirus disease 2019 (COVID-19) pandemic. Infect Control Hosp Epidemiol.

[16] Su Z, McDonnell D, Cheshmehzangi A, Li X, Maestro D, Šegalo S, Ahmad J (2021). With Great Hopes Come Great Expectations: A Viewpoint on Access and Adoption Issues Associated with COVID-19 Vaccines. JMIR Public Health Surveill.

[17] Su Z, Wen J, Abbas J, McDonnell D, Cheshmehzangi A, Li X, Ahmad J, Šegalo S, Maestro D, Cai Y (2020). A race for a better understanding of COVID-19 vaccine non-adopters. Brain Behav Immun Health.

[18] Su Z, McDonnell D, Wen J, Kozak M, Abbas J, Šegalo S, Li X, Ahmad J, Cheshmehzangi A, Cai Y, Yang L, Xiang Y (2021). Mental health consequences of COVID-19 media coverage: the need for effective crisis communication practices. Global Health.

[19] Su Z, Wen J, McDonnell D, Goh E, Li X, Šegalo S, Ahmad J, Cheshmehzangi A, Xiang Y (2021). Vaccines are not yet a silver bullet: The imperative of continued communication about the importance of COVID-19 safety measures. Brain Behav Immun Health.

[20] Djalante R, Lassa J, Setiamarga D, Sudjatma A, Indrawan M, Haryanto B, Mahfud C, Sinapoy MS, Djalante S, Rafliana I, Gunawan La, Surtiari GAK, Warsilah H (2020). Review and analysis of current responses to COVID-19 in Indonesia: Period of January to March 2020. Progress in Disaster Science.

[21] Renda A, Castro R (2020). Towards Stronger EU Governance of Health Threats after the COVID-19 Pandemic. Eur J Risk Regul.

[22] Maani N, Galea S (2020). COVID-19 and Underinvestment in the Public Health Infrastructure of the United States. Milbank Q.

[23] Ridley EJ, Freeman-Sanderson A, Haines KJ (2021). Surge capacity for critical care specialised allied health professionals in Australia during COVID-19. Aust Crit Care.

[24] Jia P, Yang S (2020). Are we ready for a new era of high-impact and high-frequency epidemics?. Nature.

[25] Jia P, Yang S (2020). Time to spatialise epidemiology in China. Lancet Glob Health.

[26] Prioritizing diseases for research and development in emergency contexts. World Health Organization.

[27] Jia P (2020). Understanding the Epidemic Course in Order to Improve Epidemic Forecasting. Geohealth.

[28] COVID-19 Dashboard by the Center for Systems Science and Engineering (CSSE) at Johns Hopkins University (JHU). Johns Hopkins University: Coronavirus Resource Center.

[29] Honigsbaum M (2019). Disease X and other unknowns. Lancet.

[30] Young PR (2020). Disease X ver1.0: COVID-19. Microbiol Aust.

[31] Simpson S, Kaufmann M, Glozman V, Chakrabarti A (2020). Disease X: accelerating the development of medical countermeasures for the next pandemic. Lancet Infect Dis.

[32] Osterholm MT (2005). Preparing for the next pandemic. N Engl J Med.

[33] Morse SS, Mazet JA, Woolhouse M, Parrish CR, Carroll D, Karesh WB, Zambrana-Torrelio C, Lipkin WI, Daszak P (2012). Prediction and prevention of the next pandemic zoonosis. Lancet.

[34] Wagar E (2016). Bioterrorism and the Role of the Clinical Microbiology Laboratory. Clin Microbiol Rev.

[35] Green MS, LeDuc J, Cohen D, Franz DR (2019). Confronting the threat of bioterrorism: realities, challenges, and defensive strategies. Lancet Infect Dis.

[36] Henderson DA (1999). The looming threat of bioterrorism. Science.

[37] Nie J, Guo N, Selden M, Kleinman A (2010). Japan's Wartime Medical Atrocities: Comparative Inquiries in Science, History, and Ethics.

[38] Tsuneishi K (2007). Unit 731 and the human skulls discovered in 1989: Physicians carrying out organized crimes. Dark medicine: Rationalizing unethical medical research.

[39] Brody H, Leonard SE, Nie J, Weindling P (2014). U.S. responses to Japanese wartime inhuman experimentation after World War II. Camb Q Healthc Ethics.

[40] Nie J (2002). Japanese doctors' experimentation in wartime China. Lancet.

[41] Bärnighausen T, Nie JB, Guo N, Selden M, Kleinman A (2010). Data generated in Japan’s biowarfare experiments on human victims in China, 1932–1945, and the ethics of using them. Japan's Wartime Medical Atrocities Comparative Inquiries in Science, History, and Ethics.

[42] Keiichi T (2005). Unit 731 and the Japanese Imperial Army's Biological Warfare Program. Asia Pac J.

[43] Mellanby K (1947). Medical experiments on human beings in concentration camps in Nazi Germany. Br Med J.

[44] Berger RL (1990). Nazi science--the Dachau hypothermia experiments. N Engl J Med.

[45] Mitscherlich A, Mielke F (1949). Doctors Of Infamy: The Story Of The Nazi Medical Crimes.

[46] McLeish C, Nightingale P (2007). Biosecurity, bioterrorism and the governance of science: The increasing convergence of science and security policy. Research Policy.

[47] (2009). 2009 UNISDR terminology on disaster risk reduction. United Nations Office for Disaster Risk Reduction.

[48] (2021). Japan: UN experts ‘deeply disappointed’ by decision to discharge Fukushima water. UN News.

[49] Convention on the Prohibition of the Development, Production and Stockpiling of Bacteriological (Biological) and Toxin Weapons and on Their Destruction. United Nations: Office for Disarmament Affairs.

[50] Jansen HJ, Breeveld FJ, Stijnis C, Grobusch MP (2014). Biological warfare, bioterrorism, and biocrime. Clin Microbiol Infect.

[51] Akerlof GA, Yellen JL (1987). Rational Models of Irrational Behavior. Am Econ Rev.

[52] Mayo E (2016). The Irrational Factor in Human Behavior. Ann Am Acad Political Soc Sci.

[53] Kahneman D, Tversky A (1979). Prospect Theory: An Analysis of Decision under Risk. Econometrica.

[54] Fernandez E, Callen A, Johnson S, Gaspar C, Kulhanek C, Jose-Bueno C (2020). Prevalence, elicitors, and expression of anger in 21st century mass shootings. Aggress Violent Behav.

[55] Koehler A, Scott RA, Davis R (2014). Surviving the dark night: the Aurora, Colorado, mass shootings. J Emerg Nurs.

[56] Bjelopera J, Bagalman E, Caldwell S, Finklea K, McCallion G (2013). Public Mass Shootings in the United States: Selected Implications for Federal Public Health and Safety Policy. Congressional Research Service.

[57] Guillemin J (2004). Biological Weapons: From the Invention of State-Sponsored Programs to Contemporary Bioterrorism.

[58] Gostin LO, Sapsin JW, Teret SP, Burris S, Mair JS, Hodge JG, Vernick JS (2002). The Model State Emergency Health Powers Act: planning for and response to bioterrorism and naturally occurring infectious diseases. JAMA.

[59] Mothershead JL, Tonat K, Koenig KL (2002). Bioterrorism preparedness. III: State and federal programs and response. Emerg Med Clin North Am.

[60] Koplan J (2001). CDC's strategic plan for bioterrorism preparedness and response. Public Health Rep.

[61] Riedel S (2004). Biological warfare and bioterrorism: a historical review. Proc (Bayl Univ Med Cent).

[62] Metropolis N, Rota G, Sharp D, Oppenheimer J (1984). I. Uncommon sense. Uncommon Sense.

[63] (2011). Timeline: How The Anthrax Terror Unfolded. NPR.

[64] Murch RS (2011). “Amerithrax”: The Investigation of Bioterrorism Using Bacillus Anthracis Spores in Mailed Letters. Encyclopedia of Bioterrorism Defense.

[65] Willman D (2011). The Mirage Man: Bruce Ivins, the Anthrax Attacks, and America's Rush to War.

[66] Hopman J, Allegranzi B, Mehtar S (2020). Managing COVID-19 in Low- and Middle-Income Countries. JAMA.

[67] Truelove S, Abrahim O, Altare C, Lauer SA, Grantz KH, Azman AS, Spiegel P (2020). The potential impact of COVID-19 in refugee camps in Bangladesh and beyond:  A modeling study. PLoS Med.

[68] Dureab F, Al-Awlaqi S, Jahn A (2020). COVID-19 in Yemen: preparedness measures in a fragile state. Lancet Public Health.

[69] Senghore M, Savi MK, Gnangnon B, Hanage WP, Okeke IN (2020). Leveraging Africa's preparedness towards the next phase of the COVID-19 pandemic. Lancet Glob Health.

[70] Garoon J, Duggan P (2008). Discourses of disease, discourses of disadvantage: a critical analysis of National Pandemic Influenza Preparedness Plans. Soc Sci Med.

[71] Fineberg HV (2014). Pandemic preparedness and response--lessons from the H1N1 influenza of 2009. N Engl J Med.

[72] Su Z, McDonnell D, Ahmad J, Cheshmehzangi A, Li X, Meyer K, Cai Y, Yang L, Xiang Y (2020). Time to stop the use of 'Wuhan virus', 'China virus' or 'Chinese virus' across the scientific community. BMJ Glob Health.

[73] Sánchez-Duque JA, Su Z, Rosselli D, Chica-Ocampo MC, Lotero-Puentes MI, Bolaños-Portilla AM, Dhawan M, Rodríguez-Morales AJ, Dhama K (2021). The ignored pandemic of public health corruption: A call for action amid and beyond SARS-COV-2/COVID-19. JEBAS.

[74] Su Z, McDonnell D, Li Y (2021). Why is COVID-19 more deadly to nursing home residents?. QJM.

[75] Oxford JS, Gill D (2018). Unanswered questions about the 1918 influenza pandemic: origin, pathology, and the virus itself. Lancet Infect Dis.

[76] Trilla A, Trilla G, Daer C (2008). The 1918 "Spanish flu" in Spain. Clin Infect Dis.

[77] Reid AH, Taubenberger JK, Fanning TG (2004). Evidence of an absence: the genetic origins of the 1918 pandemic influenza virus. Nat Rev Microbiol.

[78] Ye Z, Yuan S, Yuen K, Fung S, Chan C, Jin D (2020). Zoonotic origins of human coronaviruses. Int J Biol Sci.

[79] Kreuder Johnson C, Hitchens PL, Smiley Evans T, Goldstein T, Thomas K, Clements A, Joly DO, Wolfe ND, Daszak P, Karesh WB, Mazet JK (2015). Spillover and pandemic properties of zoonotic viruses with high host plasticity. Sci Rep.

[80] Gill P, Horgan J, Deckert P (2014). Bombing alone: tracing the motivations and antecedent behaviors of lone-actor terrorists,. J Forensic Sci.

[81] Li S (1982). The Compendium of Materia Medica.

[82] Vongkamjan K, Wiedmann M (2015). Starting from the bench--prevention and control of foodborne and zoonotic diseases. Prev Vet Med.

[83] Smith F (2020). On the hunt for the next deadly virus. National Geographic.

[84] Vanmechelen B, Rector A, Maes P (2017). Virus Hunting: Discovery of New Episomal Circular Viruses by Rolling Circle Techniques. Curr Protoc Microbiol.

[85] Olival KJ, Hosseini PR, Zambrana-Torrelio C, Ross N, Bogich TL, Daszak P (2017). Host and viral traits predict zoonotic spillover from mammals. Nature.

[86] Sharma A, Gupta G, Ahmad T, Krishan K, Kaur B (2020). Next generation agents (synthetic agents): Emerging threats and challenges in detection, protection, and decontamination. Handbook on Biological Warfare Preparedness.

[87] Noyce RS, Lederman S, Evans DH (2018). Construction of an infectious horsepox virus vaccine from chemically synthesized DNA fragments. PLoS One.

[88] Koblentz GD (2017). The De Novo Synthesis of Horsepox Virus: Implications for Biosecurity and Recommendations for Preventing the Reemergence of Smallpox. Health Secur.

[89] Pietsch B (2020). Central and Southern California have 0 percent I.C.U. capacity, in a state already low on hospital beds. The New York TImes.

[90] Blumenthal D, Fowler EJ, Abrams M, Collins SR (2020). Covid-19 - Implications for the Health Care System. N Engl J Med.

[91] Cabarkapa S, Nadjidai SE, Murgier J, Ng CH (2020). The psychological impact of COVID-19 and other viral epidemics on frontline healthcare workers and ways to address it: A rapid systematic review. Brain Behav Immun Health.

[92] Jia P (2019). Spatial lifecourse epidemiology. Lancet Planet Health.

[93] Yang S, Pan X, Zeng P, Jia P (2021). Spatial technologies to strengthen traditional testing for SARS-CoV-2. Trends Microbiol.

[94] Turing A, Braithwaite R, Jefferson G, Newman M, Copeland BJ (2004). Can Automatic Calculating Machines Be Said To Think? (1952). The Essential Turing.

[95] Shukla S, Jaiswal V (2013). Applicability of Artificial Intelligence in Different Fields of Life. Int J Sci Eng Res.

[96] Davenport T, Guha A, Grewal D, Bressgott T (2019). How artificial intelligence will change the future of marketing. J Acad Mark Sci.

[97] Liang B, Yang N, He G, Huang P, Yang Y (2020). Identification of the Facial Features of Patients With Cancer: A Deep Learning-Based Pilot Study. J Med Internet Res.

[98] Laghi A (2020). Cautions about radiologic diagnosis of COVID-19 infection driven by artificial intelligence. Lancet Digit Health.

[99] Kuziemski M, Misuraca G (2020). AI governance in the public sector: Three tales from the frontiers of automated decision-making in democratic settings. Telecomm Policy.

[100] Quer G, Radin JM, Gadaleta M, Baca-Motes K, Ariniello L, Ramos E, Kheterpal V, Topol EJ, Steinhubl SR (2021). Wearable sensor data and self-reported symptoms for COVID-19 detection. Nat Med.

[101] Zeng Z, Chen P, Lew AA (2020). From high-touch to high-tech: COVID-19 drives robotics adoption. Tourism Geographies.

[102] Tavakoli M, Carriere J, Torabi A (2020). Robotics, Smart Wearable Technologies, and Autonomous Intelligent Systems for Healthcare During the COVID‐19 Pandemic: An Analysis of the State of the Art and Future Vision. Advanced Intelligent Systems.

[103] Lin L, Hou Z (2020). Combat COVID-19 with artificial intelligence and big data. J Travel Med.

[104] Bansal A, Padappayil RP, Garg C, Singal A, Gupta M, Klein A (2020). Utility of Artificial Intelligence Amidst the COVID 19 Pandemic: A Review. J Med Syst.

[105] Keshavarzi Arshadi A, Webb J, Salem M, Cruz E, Calad-Thomson S, Ghadirian N, Collins J, Diez-Cecilia E, Kelly B, Goodarzi H, Yuan JS (2020). Artificial Intelligence for COVID-19 Drug Discovery and Vaccine Development. Front Artif Intell.

[106] Shi F, Wang J, Shi J, Wu Z, Wang Q, Tang Z, He K, Shi Y, Shen D (2021). Review of Artificial Intelligence Techniques in Imaging Data Acquisition, Segmentation, and Diagnosis for COVID-19. IEEE Rev Biomed Eng.

[107] Klein AZ, Magge A, O'Connor K, Flores Amaro JI, Weissenbacher D, Gonzalez Hernandez G (2021). Toward Using Twitter for Tracking COVID-19: A Natural Language Processing Pipeline and Exploratory Data Set. J Med Internet Res.

[108] Jia P, Yang S (2020). China needs a national intelligent syndromic surveillance system. Nat Med.

[109] Guo K, Lu Y, Gao H, Cao R (2018). Artificial Intelligence-Based Semantic Internet of Things in a User-Centric Smart City. Sensors (Basel).

[110] Guo X, Shen Z, Zhang Y, Wu T (2019). Review on the Application of Artificial Intelligence in Smart Homes. Smart Cities.

[111] Singh S, Sharma P, Yoon B, Shojafar M, Cho G, Ra I (2020). Convergence of blockchain and artificial intelligence in IoT network for the sustainable smart city. Sustainable Cities and Society.

[112] Samsung Research 6G: The next hyper connected experience for all. everything RF.

[113] Saad W, Bennis M, Chen M (2020). A Vision of 6G Wireless Systems: Applications, Trends, Technologies, and Open Research Problems. IEEE Network.

[114] Zhou Y, Liu L, Wang L, Hui N, Cui X, Wu J, Peng Y, Qi Y, Xing C (2020). Service-aware 6G: An intelligent and open network based on the convergence of communication, computing and caching. Digital Communications and Networks.

[115] Nawaz F, Ibrahim J, Awais M, Junaid M, Kousar S, Parveen T (2020). A Review of Vision and Challenges of 6G Technology. IJACSA.

[116] Alsharif MH, Kelechi AH, Albreem MA, Chaudhry SA, Zia MS, Kim S (2020). Sixth Generation (6G) Wireless Networks: Vision, Research Activities, Challenges and Potential Solutions. Symmetry.

[117] Dang S, Amin O, Shihada B, Alouini M (2020). What should 6G be?. Nat Electron.

[118] Chowdhury MZ, Shahjalal M, Ahmed S, Jang YM (2020). 6G Wireless Communication Systems: Applications, Requirements, Technologies, Challenges, and Research Directions. IEEE Open J Commun Soc.

[119] Letaief KB, Chen W, Shi Y, Zhang J, Zhang YA (2019). The Roadmap to 6G: AI Empowered Wireless Networks. IEEE Commun Mag.

[120] Kang C, Alba D, Satariano A Surging traffic is slowing down our internet. The New York Times.

[121] Yang S, Yu C, Jia P (2021). Spatiobehavioral Characteristics - Defining the Epidemiology of New Contagious Diseases at the Earliest Moment Possible. Trends Parasitol.

[122] Jia P, Yang S (2020). Early warning of epidemics: towards a national intelligent syndromic surveillance system (NISSS) in China. BMJ Glob Health.

[123] Alag S (2020). Analysis of COVID-19 clinical trials: A data-driven, ontology-based, and natural language processing approach. PLoS One.

[124] Ancochea J, Izquierdo JL, Soriano JB (2021). Evidence of Gender Differences in the Diagnosis and Management of Coronavirus Disease 2019 Patients: An Analysis of Electronic Health Records Using Natural Language Processing and Machine Learning. J Womens Health (Larchmt).

[125] Calvo RA, Milne DN, Hussain MS, Christensen H (2017). Natural language processing in mental health applications using non-clinical texts. Nat Lang Eng.

[126] Hirschberg J, Manning CD (2015). Advances in natural language processing. Science.

[127] Pestian J, Nasrallah H, Matykiewicz P, Bennett A, Leenaars A (2010). Suicide Note Classification Using Natural Language Processing: A Content Analysis. Biomed Inform Insights.

[128] Coppersmith G, Hilland C, Frieder O, Leary R (2017). Scalable mental health analysis in the clinical whitespace via natural language processing. https://ieeexplore.ieee.org/document/7897288/authors#authors.

[129] Cook BL, Progovac AM, Chen P, Mullin B, Hou S, Baca-Garcia E (2016). Novel Use of Natural Language Processing (NLP) to Predict Suicidal Ideation and Psychiatric Symptoms in a Text-Based Mental Health Intervention in Madrid. Comput Math Methods Med.

[130] Low DM, Rumker L, Talkar T, Torous J, Cecchi G, Ghosh SS (2020). Natural Language Processing Reveals Vulnerable Mental Health Support Groups and Heightened Health Anxiety on Reddit During COVID-19: Observational Study. J Med Internet Res.

[131] Orive G, Lertxundi U, Barcelo D (2020). Early SARS-CoV-2 outbreak detection by sewage-based epidemiology. Sci Total Environ.

[132] Ge Z, Song Z, Ding SX, Huang B (2017). Data Mining and Analytics in the Process Industry: The Role of Machine Learning. IEEE Access.

[133] Ansari M, Othman F, Abunama T, El-Shafie A (2018). Analysing the accuracy of machine learning techniques to develop an integrated influent time series model: case study of a sewage treatment plant, Malaysia. Environ Sci Pollut Res Int.

[134] Carrascal M, Abian J, Ginebreda A, Barceló D (2020). Discovery of large molecules as new biomarkers in wastewater using environmental proteomics and suitable polymer probes. Sci Total Environ.

[135] Guo H, Jeong K, Lim J, Jo J, Kim Y, Park JP, Kim JH, Cho KH (2015). Prediction of effluent concentration in a wastewater treatment plant using machine learning models. J Environ Sci (China).

[136] Baraniuk C (2020). Sewage monitoring is the UK's next defence against covid-19. BMJ.

[137] Poch M, Garrido-Baserba M, Corominas L, Perelló-Moragues A, Monclús H, Cermerón-Romero M, Melitas N, Jiang SC, Rosso D (2020). When the fourth water and digital revolution encountered COVID-19. Sci Total Environ.

[138] Winn Z (2020). MIT begins testing wastewater to help detect Covid-19 on campus. Massachusetts Institute of Technology.

[139] Hamisu AW, Blake IM, Sume G, Braka F, Jimoh A, Dahiru H, Bonos M, Dankoli R, Mamuda Bello A, Yusuf KM, Lawal NM, Ahmed F, Aliyu Z, John D, Nwachukwu TE, Ayeni MF, Gumede-Moeletsi N, Veltsos P, Giri S, Praharaj I, Metilda A, Bandyopadhyay A, Diop OM, Grassly NC (2020). Characterizing Environmental Surveillance Sites in Nigeria and Their Sensitivity to Detect Poliovirus and Other Enteroviruses. J Infect Dis.

[140] Zhang Q, Zhu S (2018). Visual interpretability for deep learning: a survey. Frontiers Inf Technol Electronic Eng.

[141] Forsyth D, Ponce J (2012). Computer vision: A modern approach.

[142] Lemley J, Bazrafkan S, Corcoran P (2017). Deep Learning for Consumer Devices and Services: Pushing the limits for machine learning, artificial intelligence, and computer vision. IEEE Consumer Electron. Mag.

[143] Raza K (2020). Artificial Intelligence Against COVID-19: A Meta-analysis of Current Research. Big Data Analytics and Artificial Intelligence Against COVID-19: Innovation Vision and Approach.

[144] Ozsahin I, Sekeroglu B, Musa MS, Mustapha MT, Uzun Ozsahin D (2020). Review on Diagnosis of COVID-19 from Chest CT Images Using Artificial Intelligence. Comput Math Methods Med.

[145] Zeeshan Hameed B, Patil V, Shetty D, Naik N, Nagaraj N, Sharma D (2020). Use of Artificial Intelligence-based Computer Vision System to Practice Social Distancing in Hospitals to Prevent Transmission of COVID-19. Indian J Community Med.

[146] Loey M, Manogaran G, Taha M, Khalifa N (2021). A hybrid deep transfer learning model with machine learning methods for face mask detection in the era of the COVID-19 pandemic. Measurement (Lond).

[147] Ud Din N, Javed K, Bae S, Yi J (2020). A Novel GAN-Based Network for Unmasking of Masked Face. IEEE Access.

